# A multidisciplinary approach for peritoneal carcinomatosis and bilobar liver metastases from colorectal cancer: case report and review of the literature

**DOI:** 10.1186/s12957-015-0654-y

**Published:** 2015-08-01

**Authors:** César P. Ramírez-Plaza, Francisco J. Moreno-Ruiz, José A. Pérez-Daga

**Affiliations:** General and Digestive Surgery Service, Hospital Quirón, Avenida Imperio Argentina 1, 29004 Malaga, Spain; Avenida de las Caballerizas 15, 29016 Malaga, Spain

**Keywords:** Liver metastases, Peritoneal carcinomatosis, Colorectal cancer, Cytoreductive surgery, Intraperitoneal chemotherapy, Hyperthermia

## Abstract

**Background:**

Peritoneal carcinomatosis develops in 15 % of patients with primary colorectal cancer (CRC) and in 25 % of those with recurrence. Liver metastases are also frequent and appear at some time in 35–55 % of patients with CRC. When both conditions are present and treated palliatively, the expected median survival is 5–6 months. Recent publications suggest survival is improved when R0 resection of both peritoneal and liver diseases is achieved.

**Case presentation:**

A 36-year-old woman with synchronous peritoneal and liver metastases of colorectal origin was treated with a stepwise approach consisting of initial cytoreductive surgery, minor liver resection, intraperitoneal intraoperative hyperthermic chemotherapy, adjuvant chemotherapy, right portal embolization, and finally, right hepatectomy achieving an R0 resection. The patient is alive and free of disease after 30 months of follow-up.

**Discussion:**

Patients with peritoneal carcinomatosis and liver metastases from CRC must be carefully evaluated by multidisciplinary oncological teams in order to offer the possibility of surgery to obtain an R0 resection in selected patients (especially if the peritoneal cancer index is <19 and there is resectable or potentially resectable metastatic liver disease).

## Background

Peritoneal carcinomatosis develops in 15 % of patients with primary colorectal cancer (CRC) and in 25 % of those with a recurrence. Liver metastases are also frequent and appear at some time in 35–55 % of patients with CRC. In a retrospective analysis of 5638 patients diagnosed with metastatic CRC in 1995–2010, Thomassen et al. found 8 % of cases with simultaneous liver and peritoneal diseases treated palliatively, with a median overall survival (OS) of 5 months. This poor OS has remained unchanged over the years (12 months when the best available chemotherapy was applied vs. 2.6 months when no treatment was given) [1].

Peritoneal carcinomatosis has been classically considered as a pre-terminal condition with a poor prognosis (median OS of 6 months with the best palliative treatment). Based on Sugarbaker’s initial studies, a growing number of publications have appeared in the last 15 years reporting median OS of 24 months and overall long-term survival rates of 22–49 % for selected patients treated using an aggressive surgical approach, consisting of maximum cytoreduction plus hyperthermic intraperitoneal chemotherapy (HIPEC) [2, 3]. The American Society of Peritoneal Surface Malignancies (ASPSM) has recently established 30 months as the expected median survival for patients with peritoneal carcinomatosis of colorectal origin treated at referral centers with this multidisciplinary approach [4] .

The only treatment associated with a long-term OS and eventual cure for patients with liver metastases of colorectal origin is resection with free margins, leaving sufficient remaining hepatic parenchyma (at least two hepatic segments with adequate arterial and portal supply and venous and biliary drainage). This can only be achieved in 10–15 % of cases, though it has an overall 5-year survival rate of 44–47 % and a disease-free survival rate of 30 % [5, 6]. The use of strategies that increase the resectability of liver metastases (mainly induction chemotherapy, portal embolization, and the two-stage hepatectomy approach) has allowed resection in an additional 10–15 % of patients who were previously considered to have non-resectable disease, obtaining OS rates comparable to those of patients on first-line treatment [7].

Conceptually, the presence of metastatic liver disease is considered a contraindication for the cytoreduction + HIPEC approach in patients with peritoneal carcinomatosis. Similarly, the presence of peritoneal disease precludes a curative resection of liver metastasis. However, following reports of curative surgical attempts to treat the two metastatic sites separately, the first retrospective studies have been published showing that combined surgery of peritoneal and liver diseases is feasible and has an impact on survival in selected patients. Liver resections have so far always been performed in a single procedure (before, simultaneously, or after cytoreduction + HIPEC), and though most were limited resections, segmentectomies, major hepatectomies, and local ablative therapies have also been performed.

We report the case of a patient who had simultaneous peritoneal and liver metastatic diseases and was treated at our hospital using a stepwise multidisciplinary onco-surgical approach. This approach involved a novel sequence of actions, unreported until now for these cases, by combining liver resection (minor and major), cytoreduction + HIPEC, systemic chemotherapy, and portal embolization until an R0 status was achieved.

## Case presentation

**Fig. 1:**
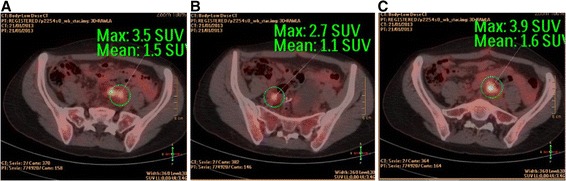
**a**-**c** PET-CT pathologic uptake in the pelvis, revealing peritoneal carcinomatosis of colorectal cancer

**Fig. 2:**
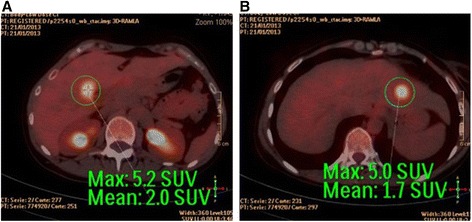
**a**-**b** PET-CT images locating the two liver metastases, the first in segment IV-B next to the right portal bifurcation and the other in segment II

**Fig. 3:**
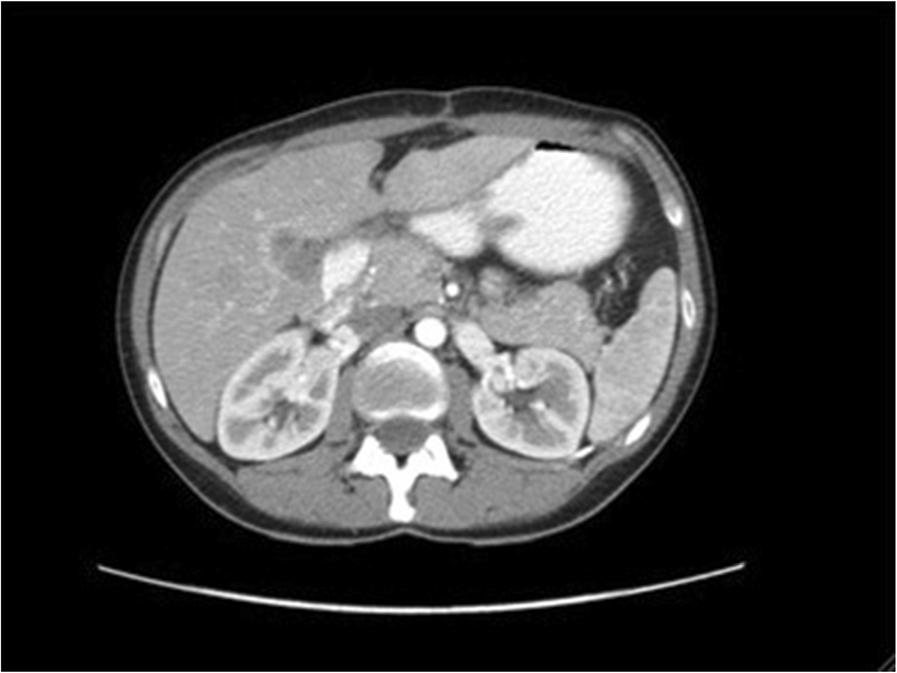
CT image showing the volume of the left liver lobe immediately after the right hepatectomy and free of disease (hypertrophy is evident)

An otherwise healthy 36 year-old woman was treated elsewhere for a perforated peri-sigmoid abscess and purulent peritonitis with sigmoidectomy and a primary mechanical colorectal anastomosis (time 0). Postoperative recovery was good, and the histopathology report described a well-differentiated adenocarcinoma (T4aNxMx, due to the absence of nodes in the surgical specimen). She was referred to the medical oncologist for palliative treatment. CEA level was 23 μU/l. A positron emission tomography-computed tomography (PET-CT) showed uptake in multiple nodes of greater than 1 cm in the pelvis (Fig. 1a–c) and two hepatic lesions suspicious for metastases (one more peripheral in segment II of 1.8 cm and the other in segment V of 2.5 cm just at the right portal bifurcation) (Fig. 2a–b). There was no extra-abdominal uptake. She came to our center to request an assessment. Her performance status on the Karnofsky scale was 100 % (no signs or symptoms of disease). Complete tumor resection was proposed and completed. This consisted of the following:

1. First surgery (week +7): a full midline laparotomy revealed a peritoneal carcinomatosis index (PCI) of 8 in the Sugarbaker classification and disease located predominantly in the pelvis [8]. As intraoperative liver ultrasound showed that the segment V lesion would require a right hepatectomy to achieve a complete R0 resection we just performed a limited segment II resection to treat the smallest liver lesion. A complete CC-0 cytoreduction (complete centripetal peritonectomy of the pelvis with a posterior pelvic-compartment exenteration and an oncological left hemicolectomy with colorectal reconstruction) was performed with intraoperative blood loss of 210 cc. This was followed by HIPEC with intraperitoneal oxaliplatin adjusted for the body surface at 43 °C for 30 min. The patient was discharged without complications on the ninth postoperative day. The biopsy confirmed the presence of peritoneal and liver metastasis with a well-differentiated non-mucinous adenocarcinoma. A K-RAS oncogene study was negative for mutation.
2. Six postoperative cycles with capecitabine, oxaliplatin, and cetuximab were given with good tolerance (weeks +12 to +26). A control PET-CT showed a complete radiological response with remission of the hepatic lesion of segment V.
3. Percutaneous right portal vein embolization (week +27). The growth of the left liver lobe after 4 weeks was 35 %, accounting for 43 % of the total hepatic volume.
4. Second surgery (week +31): an anatomical right hepatectomy was performed with a Pringle maneuver, i.e., clamping of the complete liver inflow at the hepatic hilum for 20 min, requiring transfusion of one unit of packed red cells (Fig. 3). Recovery was good, and the patient was discharged on postoperative day +8 with no complications. The histological report of the nodule was fibrosis without residual tumor cells (complete microscopic remission).

Postsurgical consolidation systemic chemotherapy was not considered necessary by the medical oncologist, and the patient is currently disease free after 30 months of follow-up.

### Discussion

No clear therapeutic action can currently be defined for patients with coexisting peritoneal carcinomatosis and liver metastasis of colorectal origin when these are the only sites of disseminated disease. The systematic review by De Cuba et al. only found a trend (with no statistical significance) towards increased OS of patients who just had peritoneal disease compared to those who had metastasis in both sites when an R0 resection was performed. They also concluded that there was no solid evidence in the literature to justify the exclusion of selected patients for resective liver surgery and cytoreduction + HIPEC [9]. The key point is patient selection in order to balance the extent of the disease with the complexity of the surgery required.

Studies on liver resection combined with cytoreduction + HIPEC for the treatment of metastatic colorectal cancer are retrospective and heterogeneous and present contradictory results (wide variation in the number of cases, centers included, type of liver resections considered, with or without ablative therapies, timing of cytoreduction + HIPEC, and techniques used to administer intraperitoneal chemotherapy). Kianmanesh et al., Chua et al., and Varban et al. reported historical series of patients with peritoneal carcinomatosis subjected to cytoreduction + HIPEC and included a subgroup in which liver resection for metastases was also performed, finding no differences in OS between the groups [10–12]. Similarly, Elias et al. in a French multicenter study including 523 patients with peritoneal carcinomatosis of colorectal origin (95 % of cases having optimal cytoreduction, CC-0 or CC-1) did not find that the presence of liver metastasis (*n* = 77) and its simultaneous resection had a negative impact on OS (5-year OS of 21 vs. 27 %) [13]. However, Glehen et al., in a European multicenter review of 506 cases of peritoneal carcinomatosis of colorectal origin, identified liver metastases as a negative prognostic factor of survival (16.8 vs. 20.4 months), but optimal cytoreduction was only achieved in 74.5 % of the patients [14]. In all these studies, the administration of systemic adjuvant chemotherapy after surgery proved to be a favorable prognostic factor for survival. Maggiori et al. published a retrospective cohort study comparing a historic series from the Gustave-Roussy Institute of 37 cases treated for simultaneous peritoneal and liver metastases of colorectal origin with a group of 61 controls who had been treated for peritoneal disease as the only form of metastasis during the same period (Table 1). A peritoneal cancer index (PCI) >12 and simultaneous liver and peritoneal surgery were negative predictive factors for survival. Thus, they established the first prognostic stratification based on a logistic regression model for these patients: those with a PCI <12 and absence of liver metastases, median survival of 76 months; PCI <12 and presence of one or two liver metastases, 40 months; and finally, PCI >12 or more than two liver tumors, 27 months [15]. The most recent reviews suggest no benefit in OS by performing radical surgery accompanied by HIPEC in patients with a PCI >19. However, as a few selected patients with a PCI = 12–19 or >2 liver metastases can benefit from a combined radical approach patients should be individualized. In our patient, who only had two identified liver lesions and a preoperative PCI <12 estimated from the CT and PET-CT images, we considered a strategy to achieve an R0 status as the best option. Our limiting factor was the complex site of the right liver lesion (requiring a right hepatectomy), which led us to design a multidisciplinary, step-by-step approach using all the medical, technical and radiological resources, and information from the liver and peritoneal biopsies.

**Table 1:** Major published series of patients undergoing surgery for coexisting peritoneal carcinomatosis and liver metastases from colorectal cancer

Author	Year	*n* (peritoneal + liver/total)	OS (peritoneal + liver/only peritoneal)	Observations
Kianmanesh et al.	2007	37.2 % (16/43)	36 vs. 35.3 months	No differences between groups
				Only 70 % of patients having CC-0 or CC-1 surgery
Chua et al.	2009	29.1 % (16/55)	65 vs. 68 %, 2 years	No differences between groups
				Group of combined liver and peritoneal, lower PCI (8 vs. 12)
Varban et al.	2009	9.9 % (14/142)	23 vs. 15.8 months	No differences between groups
			43.3 vs. 36.8 %, 2 years	Median number and size of liver metastasis: 1 (1–7) and 3 cm (0.4–12)
Elias et al.	2009	14.72 % (77/523)	21 vs. 27 %, 5 years	Multicentric study
				No differences between groups
Glehen et al.	2004	12.15 % (61/502)	16.8 vs. 20.4 months	Liver metastasis as negative predictive factor for OS (*p* = 0.008)
Maggiori et al.	2013	37 vs. 60	40 vs. 66 %, 3 years	Median OS 40 months for PCI <12 and 1 or 2 liver metastases
			32 vs. 49 months	

The management of the liver metastases in series of Table 1 does not correspond with current modern criteria for optimizing OS after liver surgery because there are R1 or R2 resections, ablative treatments mixed with surgery, and different types and extension of liver resections. Radical R0 liver resection is the most important known prognostic factor in surgery of liver metastasis and has a 5-year OS of 32 % [16, 17]. Patients subjected to ablative therapies, either as single therapy or complementary to hepatectomy, cannot be considered in the same group as those who just undergo resection. Series with long follow-up periods have shown that they are not comparable treatments because local recurrences are greater and nearly universal (1.7–66.7 % for ablative vs. 1.2–10.4 % for resection) [18]. On the other hand, the extension of the hepatectomy (major or major extended vs. minor), the need for perioperative transfusion (which is also dose-dependent), and postoperative complications (particularly infectious morbidity and postoperative liver failure) are considered negative prognostic factors for survival by increasing the risk of recurrence, which occurs exclusively in the liver in almost half the cases (43.2 %) [6, 19–21].

The initial studies by Paul Brousse demonstrated that up to 12.5 % of patients with liver metastases initially considered as non-resectable were able to be rescued secondarily for complete radical surgery when they were subjected to systemic chemotherapy with a chronomodulated infusion (5-FU + Leucovorin + Oxaliplatin or Irinotecan), with 5- and 10-year disease-free survival rates of 22 and 17 %, respectively [22]. This “neoadjuvant” chemotherapy has also been applied, in historic series, to patients with resectable liver metastases, in order to minimize the extension of the hepatectomy required to obtain curative resections (thus decreasing morbidity and mortality), eliminate potential extra-hepatic micro-metastasis, evaluate chemosensitivity (identifying and discarding patients for surgery who progress during systemic chemotherapy), and finally to evaluate the histological regression response, which is known to be a positive prognostic factor of survival. The analysis of long-term OS in EORTC 40983, a prospective randomized multicenter trial that compared patients with four or less resectable liver metastases subjected to systemic chemotherapy before and after surgery or only to surgery, found no difference (51.2 vs. 47.8 % at 5 years), despite the fact that the initial results indicated improved disease-free survival for the first group (42.4 vs. 33.2 %) [23]. Thus, although it has not been possible to demonstrate the benefits in terms of survival of neoadjuvant systemic chemotherapy for resectable liver disease with a high level in evidence-based medicine, its use seems appropriate because it enables selection of patient subgroups with a better prognosis, and for some patients in progression, it avoids surgery (which will not contribute in the natural course of the disease). In our case, we recommended chemotherapy after the first peritoneal surgery as an adjuvant option and to evaluate the response of the remnant liver during this period, in order to obtain biologic information about the disease.

Patients with multiple liver metastases treated with systemic chemotherapy usually require more than one liver surgery procedure, as do patients with liver lesions due to different forms of toxicity (non-alcoholic steatosis and sinusoidal obstruction syndrome, mainly associated with oxaliplatin and irinotecan). Portal embolization and two-stage hepatectomy have been the strategies that have helped liver surgeons to achieve more R0 resections in the most technically complex and extreme cases, those in which it is estimated that there is a residual preoperative volume of <25 % of healthy liver, or <30–40 % with baseline liver steatosis or damage by chemotherapy. Portal embolization enables safer liver resection and, furthermore, increases the number of patients susceptible to major hepatectomy by 20 %, with a 5-year survival rate of around 40 % (similar to that obtained without portal embolization) [24]. The two-stage approach allows an R0 resection in a multifocal metastatic liver in two operations that was previously untreatable in one-time surgery. The first time is usually for minor liver surgery (limited resections or of less than three segments), and the second time is reserved for major surgery, with portal embolization and systemic chemotherapy support usually given in the interval between the procedures. A recently published systematic review reported a median survival of 37 months, with a disease-free survival of 20 % at 3 years for patients completing the two-stage liver approach [25]. In our case, the accumulated dose of chemotherapy and the need for major liver resection warranted optimizing the liver surgery by combining these two strategies.

## Conclusions

1. To date, no randomized level 1 evidence is available (nor is it likely to be) to consider surgery as the standard or recommended treatment for patients with coexisting peritoneal and liver metastases from colorectal cancer. The relevant medical literature is based on heterogeneous, retrospective, low-volume series.
2. Optimal cytoreduction + HIPEC and liver resection represent the best therapeutic option for selected patients with low-volume peritoneal carcinomatosis and liver metastases from colorectal cancer. If an R0 status can be achieved, the patient will have a clear benefit regarding overall survival.
3. Portal embolization and two-stage hepatectomy are strategies with proven efficacy in the treatment of liver metastases and can be integrated into the multidisciplinary approach for these patients in order to increase the possibility of an R0 resection and decrease morbidity related to the liver resection.
4. Systemic chemotherapy has a positive impact on overall survival in patients treated surgically for peritoneal and liver metastatic colorectal disease and must always be used in the multi-step strategies considered.
5. These patients must be referred to and treated at centers with surgeons and medical teams experienced in cytoreduction + HIPEC and surgery for liver metastases.

## Consent

Written informed consent was obtained from the patient for the publication of this case report and the accompanying images. A copy of the written consent is available for review by the editor-in-chief of this journal if required.

## Abbreviations

ASPSM: American Society of Peritoneal Surface Malignancies
CRC: colorectal cancer
CT: computed tomography
HIPEC: hyperthermic intraperitoneal chemotherapy
OS: overall survival
PCI: peritoneal carcinomatosis index
PET: positron emission tomography
